# Efficient Green Synthesis of (Fe_3_O_4_) and (NiFe_2_O_4_) Nanoparticles Using Star Anise (*Illicium verum*) Extract and Their Biomedical Activity against Some Cancer Cells

**DOI:** 10.3390/ma15144832

**Published:** 2022-07-11

**Authors:** Noha Al-Qasmi, Fahad A. Almughem, Somayah J. Jarallah, Amani Almaabadi

**Affiliations:** 1 Chemistry Department, Faculty of Science, Taif University, Al Hawiyah, P.O. Box 11099, Taif 21944, Saudi Arabia; 2 National Center for Biotechnology, Life Science and Environment Research Institute, King Abdulaziz City for Science and Technology (KACST), Riyadh 11442, Saudi Arabia; falmughem@kacst.edu.sa (F.A.A.); sjarallah@kacst.edu.sa (S.J.J.)

**Keywords:** green synthesis, magnetite, spinel NiFe_2_O_4_, star anise, co-precipitation

## Abstract

Magnetite Fe_3_O_4_ and spinel (2:1) and (4:1) NiFe_2_O_4_ magnetic nanoparticles (MNPs) were prepared by simple and affordable co-precipitation methods using an extract of star anise (*Illicium verum*) as a green reducing agent. The morphology and chemical composition of these MNPs were confirmed by field-emission scanning electron microscopy, energy-dispersive X-ray spectroscopy, UV–visible spectroscopy, and X-ray diffraction (XRD). The synthesized magnetite Fe_3_O_4_ and spinel (2:1) and (4:1) NiFe_2_O_4_ MNPs were in the size range of 0.1–1 µm. The MNPs had irregular clustered platelets (magnetite Fe_3_O_4_) and pyramidal structures (spinel (2:1) and (4:1) NiFe_2_O_4_ NPs). The average sizes of the synthesized magnetite Fe_3_O_4_, and spinel (2:1) and (4:1) NiFe_2_O_4_ MNPs calculated using XRD analysis were 66.8, 72.5, and 72.9 nm, respectively. In addition to the characteristic absorption peaks of magnetite Fe_3_O_4_, those of spinel (2:1) and (4:1) NiFe_2_O_4_ MNPs were detected at ~300–350 nm and ~700 nm, respectively. Overall, the results of this study indicate that the synthesized magnetite Fe_3_O_4_, and spinel (2:1) and (4:1) NiFe_2_O_4_ MNPs showed high biomedical activities against liver carcinoma cells and non-small lung adenocarcinoma cells.

## 1. Introduction

Magnetic nanoparticles (MNPs) have been extensively investigated owing to their interesting properties, such as excellent magnetic activity, chemical and thermal stability, high surface-area-to-volume ratio, good adsorption behavior, and photocatalytic activity [1,2,3,4]. However, ferrite in the form of magnetite (Fe_3_O_4_), maghemite (γ-Fe_2_O_3_), and doped-ferrite have particularly received a large amount of attention [5,6,7]. Generally, based on the crystal structure, ferrite nanoparticles are classified as hexagonal (MFe_12_O_19_), garnet (M_3_Fe_5_O_12_), or spinel (MFe_2_O_4_) structures, where M is a transition metal cation such as Ni, Mg, Co, Cu, or Zn [8,9]. MNPs have potential uses in a wide variety of applications based on their electrochemical and antimicrobial activity, such as organic catalysis, photocatalysis, fuel cells, electronic devices, water remediation, drug delivery, and cell therapy [10,11,12,13]. The conventional physical and chemical methods that are used to synthesize MNPs are co-precipitation, thermal decomposition, sonochemical, sol–gel, hydrothermal, and chemical combustion methods [14,15,16,17]. In recent decades, the limitations of these methods have been clarified, including long processing times, high cost, use of hazardous chemical compounds, and the release of toxic reagents into the environment [18,19].

Currently, green nanotechnology is attracting significant scientific attention owing to its high potential for addressing environmental challenges as it is inexpensive, safe, and eco-friendly [20,21]. Therefore, improved protocols for the green synthesis of MNPs using natural extracts and microorganisms have been reported. Prokaryotes (e.g., bacteria, algae, and fungi) produce a large number of inorganic materials as by-products because of their huge biodiversity [22]. In addition, the leaves, roots, seeds, and flowers of plants contain diverse phenolic compounds that can reduce metal ions to metal nanoparticles (NPs) simply and rapidly [23,24]. Therefore, the analysis of such compounds for MNP biosynthesis may be of use more broadly for other applications as well. In addition, there is potential for MNPs to have biotechnological and medical applications [25,26].

The main purpose of the current research was to develop a green method for the synthesis of two types of MNPs: magnetite (Fe_3_O_4_) and spinel nickel ferrite [(2:1) and (4:1) NiFe_2_O_4_], via a simple, rapid, economical and effective co-precipitation method. Star anise (*Illicium verum*) extract was used as an eco-friendly reducing agent for the reduction of metal ions to metal nanoparticles. To the best of our knowledge, this is the first time that the preparation of magnetite Fe_3_O_4_, and spinel (2:1) and (4:1) NiFe_2_O_4_ MNPs using star-anise extract has been reported. The synthesized MNPs were characterized using field-emission scanning electron microscopy (FESEM), energy-dispersive X-ray spectroscopy (EDS), UV–visible spectroscopy (UV–vis), and X-ray diffraction (XRD). Once characterized, the cytotoxicity effect of the MNPs against liver carcinoma cells and non-small lung adenocarcinoma cells was examined as an example of a biomedical application.

## 2. Experimental Section

### 2.1. Chemicals

Analytical-grade iron chloride (FeCl_3_·6H_2_O, FeCl_2_·4H_2_O), and nickel chloride (NiCl_2_. 6H_2_O) were obtained from Sigma-Aldrich and used without additional purification. All chemicals and plant extracts were prepared in this investigation using double-distilled water as the solvent.

### 2.2. Preparation of the Star Anise Extract

Star anise (*Illicium verum*) was purchased from a local market. To prepare the extract of star anise, we used the same procedure as reported previously [27]. The extract was filtered and centrifuged at 15,000 rpm before being used for the preparation of MNPs.

### 2.3. Synthesis of the Magnetic Nanomaterials

Magnetite Fe_3_O_4_, and spinel (2:1) and (4:1) NiFe_2_O_4_ MNPs were synthesized by green co-precipitation methods. First, FeCl_3_·6H_2_O and FeCl_2_·4H_2_O with 1:2 molar proportions were dissolved in 100 mL of double-distilled water. The reaction mixture was then boiled for 10 min at 60 °C on a hot plate with vigorous stirring. Next, 10 mL of the star-anise extract was added to the reaction mixture. The color changed from light to darkish brown, indicating the production of Fe_3_O_4_ MNPs. Then, the solution was dried in an oven for 24 h at 80 °C. Subsequently, the obtained powder was calcined at 700 °C for 2 h [28]. Second, the spinel NiFe_2_O_4_ MNPs with (2:1) and (4:1) ratio of (NiCl_2_·6H_2_O: FeCl_3_·6H_2_O) were synthesized following the same above procedure.

### 2.4. Instrumentation and Characterization

The morphology and elemental composition of the MNPs were studied using FESEM and EDS measurements (JEOL JSM-7600 F Technologies Ltd., Raleigh, NC, USA). A Scanting XDS 2000 diffractometer equipped with a Cu Kα radiation source was used to obtain the XRD patterns of the MNPs. The spectroscopic analysis of the MNPs was performed using a UV/VIS/NIR spectrometer (Lambda 750, Parkin Elmer).

### 2.5. In Vitro Cytotoxicity Evaluation

In vitro cytotoxicity evaluation of the three different types of MNPs was performed against liver carcinoma cells (Hep G2, ATCC number HB-8065) and non-small lung adenocarcinoma cells (A549, ATCC number CCL-185). All cell products were purchased from the American Type Culture Collection (ATCC). Living cellular models were used between passages 12–27. Dulbecco’s modified Eagle’s medium (DMEM) supplemented with 10% (*v*/*v*) fetal bovine serum (FBS), streptomycin (100 μg/mL), and penicillin (100 U/mL) were used to maintain the two cell lines.

The cellular viability of Hep G2 and A549 cells following the application of the MNPs was assessed by measuring the cellular metabolic activity using MTS assay kit (CellTiter 96 Aqueous One Solution Cell Proliferation Assay, Promega, Madison, WI, USA). Following the cell confluency, cells were detached from the flask using trypsin, then counted with the trypan blue exclusion test, and seeded at a seeding density of 1.5 × 104 cells/well into 96-well plates. The samples were then incubated overnight in a humidified 5% CO_2_ cell culture incubator at 37 °C. The next day, 100 µL samples of increasing concentrations of the tested MNPs (15.62–1000 µg/mL) were incubated with the two human cancerous cell types for 24 h. The cells were incubated with only DMEM, or with Triton X-100, as the positive and negative controls, respectively. The investigated nanoparticles were aspirated from the wells, 100 μL of DMEM was added, and then 20 μL of the MTS reagent was added to each well. Thereafter, cells were covered with aluminum foil and incubated for 2–3 h at 37 °C. A Cytation 3 absorbance microplate reader (BioTek Instruments Inc., Winooski, VT, USA) was used to measure MTS absorbance at 490 nm. The percentage of viable cells was calculated using the following equation:

 Cell viability (%)=(S−T)/(H−T)×100

where S is the absorbance of the cells treated with the MNPs, H is the absorbance of the cells treated with DMEM (positive control), and T is the absorbance of the cells treated with Triton X-100 (negative control).

## 3. Results and Discussion

### 3.1. Morphology

**Figure 1:**
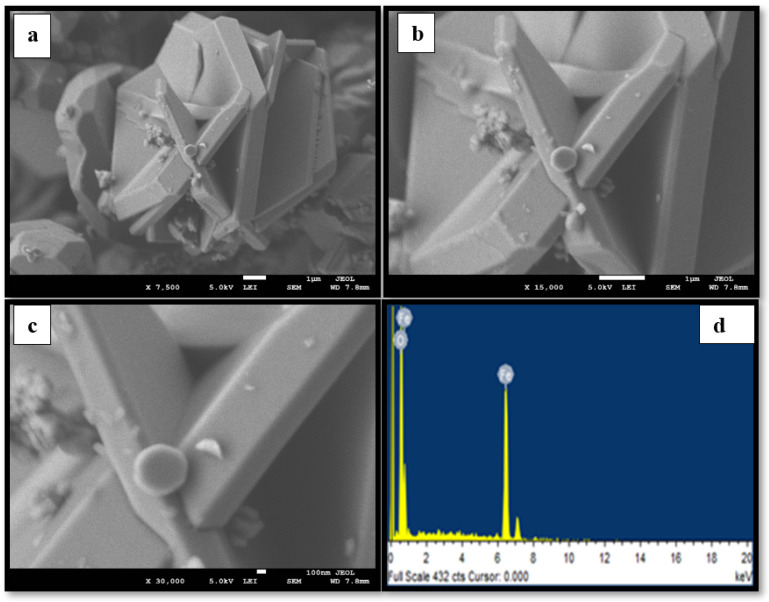
Representative FESEM images (**a**–**c**) at different magnifications of the synthesized Fe_3_O_4_ NPs and (**d**) EDS.

**Figure 2:**
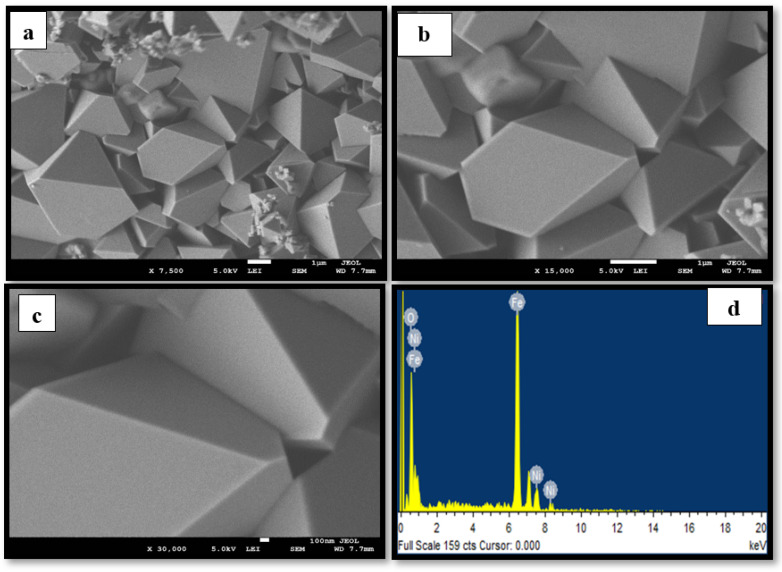
Representative (**a**–**c**) FESEM images at different magnifications and (**d**) EDS spectrum of the synthesized spinel (2:1) NiFe_2_O_4_ MNPs.

**Figure 3:**
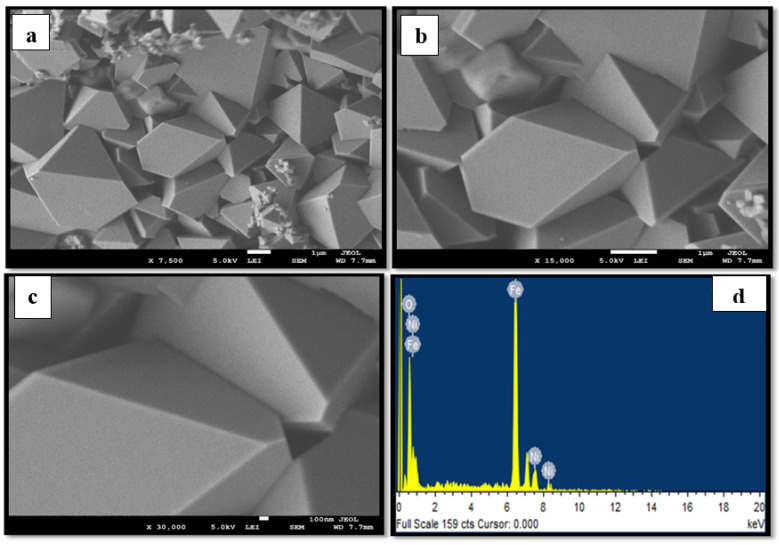
Representative (**a**–**c**) FESEM images at different magnifications and (**d**) EDS spectrum of the synthesized spinel (4:1) NiFe_2_O_4_ MNPs.

Figure 1a–c, Figure 2a–c and Figure 3a–c display the FESEM images of the MNPs synthesized using the star-anise extract. Figure 1a–c shows that the magnetite Fe_3_O_4_ MNPs comprised irregular clusters of overlapping platelets. However, Figure 2a–c and Figure 3a–c clearly depict the distinct pyramidal shapes of both spinel (2:1) and (4:1) NiFe_2_O_4_ MNPs. Therefore, the precursor metallic salts did not have a significant effect on the morphology of the spinel (2:1) and (4:1) NiFe_2_O_4_ MNPs. It is possible that the highly agglomerated shapes might be due to the magnetic properties of the MNPs [29]. The size of the MNPs was determined to be in the range of 0.1–1 µm.

The EDS spectra of the MNPs fabricated using star-anise extract are shown in Figure 1d, Figure 2d and Figure 3d. In the case of magnetite Fe_3_O_4_ (Figure 1d), strong signal peaks at 6.2 keV and 0.6 keV for iron and at 0.5 keV for oxygen were observed. The presence of iron and oxygen verified the formation of magnetite Fe_3_O_4_ MNPs [30]. Typical peaks of nickel, iron, and oxygen were noticed in the EDS spectra for the (2:1) and (4:1) spinel MNPs (Figure 2d and Figure 3d). However, it is clear that the use of star-anise extract via simple co-precipitation forms highly crystalline Fe_3_O_4_, and spinel (2:1) and (4:1) NiFe_2_O_4_ MNPs.

### 3.2. UV Spectra

**Figure 4:**
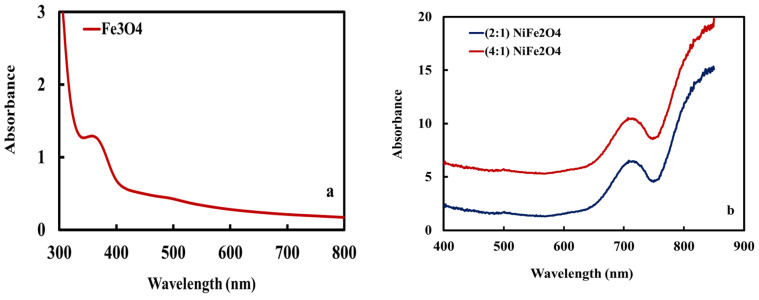
UV spectra of the synthesized (**a**) Fe_3_O_4_ and (**b**) spinel (2:1) and (4:1) NiFe_2_O_4_ MNPs.

The electronic transitions of the MNPs during the chemical reaction between the extract of star anise and precursor metallic salts were studied using UV–vis spectrophotometry in the range of 200–800 nm. The spectrum of the Fe_3_O_4_ MNPs has a strong peak at approximately 300–350 nm [31] (Figure 4a), while this peak shifted to ~700 nm for spinel (2:1) and (4:1) NiFe_2_O_4_ MNPs (Figure 4b) [32]. The clear absorption peaks indicate that the synthesized nanoparticles were stable and well dispersed in the solution.

### 3.3. Crystal Structure

**Figure 5:**
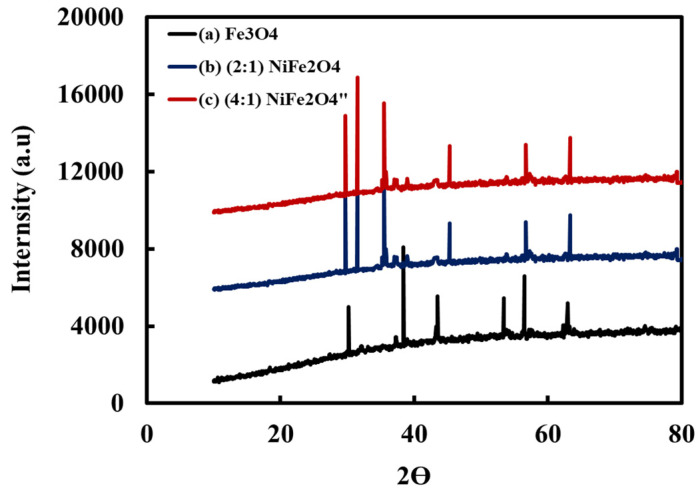
XRD spectra of the synthesized Fe_3_O_4_, and spinel (2:1) and (4:1) NiFe_2_O_4_ MNPs.

Figure 5 displays the XRD spectra of the synthesized (a) magnetite Fe_3_O_4_ and (b) spinel (2:1) NiFe_2_O_4_ and (c) (4:1) NiFe_2_O_4_ MNPs. Intense reflection peaks of the synthesized magnetite Fe_3_O_4_ MNPs were observed at 2θ = 30.8, 38.5, 43.7, 53.7, 56.5, and 62.5° of magnetite Fe_3_O_4_ [33], as shown in Figure 3a. Scherrer’s equation D = 0.9λ/β cosθ was used to calculate the average crystallite size (D), where β is the full width at half maximum (FWHM) line broadening of the most intense peak, K is the Scherrer constant, θ is Bragg’s angle, and λ is the X-ray wavelength. This analysis gave values of 66.8 nm.

The diffraction patterns of spinel (2:1) and (4:1) NiFe_2_O_4_ MNPs are shown in Figure 5 (line a,b,c), respectively. The peaks are intense and sharp, indicating the excellent crystallinity of the synthesized MNPs. Peaks were observed at 2θ values of 29. 7, 31.5, 35.5, 45.3, 56. 7, and 63.2° of the spinel NiFe_2_O_4_ structure [34]. No additional peaks were observed, indicating that the synthesized MNPs were of high purity. In addition, the crystallite size was determined using Scherrer’s formula as 72.5 and 72.9 nm.

### 3.4. In Vitro Cytotoxicity

The in vitro cytotoxicity assessment of the applied MNPs is an essential step toward biomedical application. In this experiment, increased concentrations of the MNPs were tested against Hep G2 and A549 cell lines to define the optimal concentrations that do not cause cytotoxic effects to living tissue and to identify the safety of MNPs for further studies.

Liver carcinoma cells were used because it is known that the liver is the main site for the metabolism for chemicals and food. Whether the medication is taken orally and then crosses the digestive system to the blood circulation system or whether it is taken by intravenous administration, it should pass through the liver. Furthermore, many chemicals should be metabolized through the liver before they are excreted. Either the chemical compound or the metabolite could be toxic to the liver. Therefore, the liver is one of the main organs that needs to be safe during the administration of any chemicals. Cancer in hepatic cells is ranked in fourth place for causing death for cancer-related deaths [35]. Moreover, the liver has a vital role in immunological reaction and inflammation due to its unique structure and function [35].

**Figure 6:**
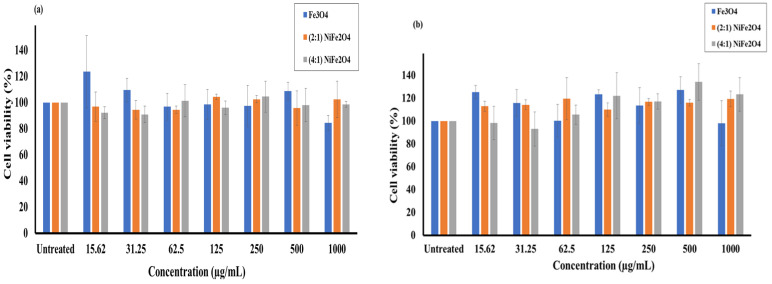
Cell viability of the synthesized Fe_3_O_4_, and spinel (2:1) and (4:1) NiFe_2_O_4_ NPs after their incubations for 24 h with (**a**) Hep G2 and (**b**) A549 cell lines. The data are the result of MTS assay expressed as cell viability (%) and presented as the mean ± SD (*n* = 3).

Figure 6a represents the effect of magnetite Fe_3_O_4_, spinel (2:1) and (4:1) NiFe_2_O_4_ NPs with different base concentrations on the cellular metabolic activity of Hep G2 cell line using MTS assay after a 24 h incubation time. The results showed high metabolic activity of all the applied nanoparticles comparable to the positive control with no observable effect of the tested nanoparticles on the cell viability. High viability of the human cancerous cells was achieved at all concentrations applied even at the maximum concentration used (1000 µg/mL). The level of viability of the highest and the lowest concentrations used was almost comparable.

Figure 6b demonstrates the percentage of cell viability of A549 cells following the application of tested MNPs and incubation for 24 h. It should be noted that high cellular metabolic viability and low cytotoxicity were obtained at all tested concentrations in all types of nanoparticles used even at the highest concentration used (1000 µg/mL). The results showed no effect of all applied doses on the cell viability as its level was nearly similar at the lowest and highest concentration applied, 15.62 and 1000 µg/mL, respectively.

The in vitro evaluation of the magnetite Fe_3_O_4_, and spinel (2:1) and (4:1) NiFe_2_O_4_ NPs showed low cytotoxicity effects on Hep G2 and A549 cells for the range of concentrations determined in the MTS assay, suggesting these MNPs as a useful bio-nanomaterial for medical applications.

## 4. Conclusions

Using a simple, rapid, inexpensive, and green co-precipitation approach, magnetite Fe_3_O_4_ and spinel (2:1) and (4:1) NiFe_2_O_4_ MNPs were successfully synthesized in this study. The star anise extract was used as an environmentally friendly reducing agent instead of a highly toxic chemical reagent. According to the findings, the synthesized MNPs were found to be extremely crystalline and pure. FESEM measurements revealed that the particle sizes of the MNPs were in the range of 0.1–1 µm. Furthermore, according to XRD analysis, the sizes of the prepared MNPs were 66.8, 72.5, and 72.9, respectively. The MNPs fabricated with the star-anise extract could be used as cancer treatments due to excellent biological activity against liver cancer and non-small lung adenocarcinoma cells.

## Data Availability

Data are contained within the article.
